# Alkaloids mediated regulation of microRNA in cancer: molecular insights and emerging therapeutic strategies

**DOI:** 10.3389/fonc.2026.1863217

**Published:** 2026-06-05

**Authors:** Syed Gulfishan, Marya Ahsan, Md Ali Mujtaba, Pratibha Pandey, Meenakshi Verma, Sorabh Lakhanpal, Shivani Sharma, Mir Waqas Alam, Fahad Khan

**Affiliations:** 1 Department of Biomedical Sciences, Division of Pharmacology, College of Medicine, King Faisal University, Al-Ahsa, Saudi Arabia; 2 Department of Pharmacology, College of Medicine, Imam Mohammad Ibn Saud Islamic University (IMSIU), Riyadh, Saudi Arabia; 3 Center for Health Research, Northern Border University, Arar, Saudi Arabia; 4 University Centre for Research and Development, Chandigarh University, Mohali, Punjab, India; 5 School of Pharmaceutical Sciences, Lovely Professional University, Phagwara, Punjab, India; 6 Uttaranchal Institute of Technology, Uttaranchal University, Dehradun, Uttarakhand, India; 7 Department of Physics, College of Science, King Faisal University, Al-Ahsa, Saudi Arabia; 8 Department of Community Medicine, Saveetha Medical College and Hospitals, Saveetha Institute of Medical and Technical Sciences, Chennai, Tamil Nadu, India

**Keywords:** alkaloids, apoptosis, cancer, miRNA, natural compounds

## Abstract

Cancer, the second leading cause of death worldwide, has prompted extensive research to uncover its mechanisms and identify effective prognostic and therapeutic strategies. Deregulation of microRNAs (miRNAs) contributes to the abnormal activation of tumorigenic processes and repression of tumor-suppressive mechanisms across various cancer types. Plants biosynthesize a wide range of structurally diverse secondary metabolites with outstanding medicinal applications. Despite notable advancements in cancer therapeutics, the current anticancer drugs and treatment approaches have not achieved complete effectiveness. The literature indicates that alkaloids and their derivatives possess strong anticancer potential and have emerged as promising pharmacological agents. Numerous alkaloids screened from herbal (medicinal) sources have demonstrated significant antiproliferative and anticancer effects against several cancer types. Numerous anticancer drugs derived from alkaloids, including vindesine, vinblastine, vincristine, and vinorelbine, have been developed and are widely used in cancer therapeutics. Research has also shown that the alkaloid regulates miRNA expression, thereby inhibiting cancer cell invasion and migration, and leading to apoptosis induction. This review comprehensively evaluated the role of alkaloids in regulating miRNA expression and their downstream targets involved in cancer progression, metastasis, and chemoresistance in various carcinomas. We highlight how miRNAs function as key regulators and mediators of the anticancer mechanisms of these natural compounds. Furthermore, we have also summarized the potent role of different types of miRNAs in cancer progression and development.

## 1 Introduction

Cancer still continues to pose a serious global public health challenge. Despite considerable progress in medical research and treatment methods, cancer continues to exert a substantial impact on populations and constitutes a key barrier to increasing life expectancy in the twenty-first century (1). As per the World Health Organization, about 9.6 million individuals died from malignancy globally in 2018 (2). The selection of an appropriate treatment approach largely depends on factors such as cancer type, disease stage, and the extent of progression within the body. In clinical practice, many patients receive treatment through a multimodal approach, which involves combining several therapeutic modalities, such as surgery, chemotherapy, and radiation therapy to improve overall treatment effectiveness (3). Because cancer treatments often cause side effects and the disease can return after therapy, researchers have been looking for adjunctive and supportive treatment options to improve clinical outcomes. In recent years, growing attention has been given to natural compounds obtained from medicinal plants (4–6). These substances may be used in different forms, such as purified or synthesized compounds, nano-encapsulated preparations, or simple plant extracts (7, 8). Many of these plant-derived compounds show biological activities that may help in cancer management. These bioactive components might influence numerous cellular processes, including signaling pathways PI3K/AKT, MAPK, and NF-κB, which are highly associated with cancer development. These dysregulated pathways further trigger apoptosis, ferroptosis, autophagy, or cell cycle growth arrest. In addition, these compounds often exhibit antioxidant and anti-inflammatory properties, which may further enhance their therapeutic potential (9, 10).

Bioactive compounds extracted from plants such as alkaloids, flavonoids, terpenoids, lignans, saponins, phenolic compounds, and glucosides have attracted considerable scientific interest due to their diverse range of biological activities (11). These compounds help in protecting normal cells from DNA damage, making them promising candidates for targeted approaches to cancer prevention (12–14). Many alkaloids show biological activity by interfering with essential cellular processes. For example, inhibition of the enzyme topoisomerase, results in blocking the cell division and leads to suppression of cancer cell growth (15). Because of these properties, alkaloids have been widely studied and used in the development of anti-inflammatory, antibacterial, and anticancer medications (16). Plant-derived alkaloids therefore play a significant role in research aimed at preventing or suppressing cancer development.

Alkaloids influence several cellular and molecular pathways involved in carcinogenesis, including abnormal cell growth, regulation of the cell cycle, and signaling pathways related to metastasis. Because of these anticancer activities, they are considered as key bioactive components in the development of novel anticancer therapeutic agents (17, 18). These plant derived compounds target cancerous cells via multiple biological mechanisms, such as altered expression of miRNAs, molecules that play a crucial role in regulating gene expression (19).

By influencing the translation of their target messenger RNAs (mRNAs), microRNAs (miRNAs) are able to exert control on the expression of genes. Because of this function, they play a significant part in the regulation of a variety of signaling pathways that occur within cells. The activity of miRNAs can be controlled at different levels, including epigenetic, transcriptional, and post-transcriptional regulation, which allows them to influence the effects of certain medications (20). For example, the anticancer drug paclitaxel has been reported to increase the expression of miR-512-3p, which targets the FADD-like apoptosis regulator (CFLAR or c-FLIP). This interaction can promote apoptosis in liver cancer cells (21). miRNAs have the potential to either promote or repress the development of cancer (22, 23). This is dependent on the genes that they regulate as well as the context of the cell. Abnormal regulation of miRNAs can also affect several biological processes, including apoptosis, autophagy, stem cell characteristics, drug transport, and epithelial mesenchymal transition (EMT). Through these mechanisms, deregulated miRNAs can affect how tumor cells respond to anticancer therapies by regulating multiple signaling pathways involved in cancer progression (24, 25).

Comprehensive understanding of the underlying molecular mechanism may help in the identification of novel therapeutic targets and contribute to the optimization of existing anticancer treatment strategies. Our aim is to review the role of miRNAs in controlling and mediating the anticancer activities of alkaloids used in cancer therapy. Additionally, it highlights recent advances in this field to facilitate the identification of promising anticancer compounds with well-defined molecular targets.

## 2 Methodology

A comprehehsive review of literature search was carried out to evaluate the significant anticancer effects of alkaloids on miRNA expression across various cancer types. A systematic search of the relevant articles published up to March 2026 was conducted across electronic databases, including Web of Science, PubMed, Scopus, sciendirect, and Google Scholar, using keywords such as alkaloids, microRNA (miRNA), tumor progression, natural compounds, plant-derived compunds, and cancer therapy. Research demonstrating the anticancer effects of plant-derived alkaloids through miRNA targeting and supporting this findings with *in vitro*, *in vivo*, or clinical models was considered for inclusion in the study. Exclusion criteria included literature that was not related to alkaoids, research using synthetic analogs of alkaloids, studies lacking adequate experimental analysis, book chapters, conference proceedings, and editorials.

## 3 Alkaloids as anticancer agent

Alkaloids are organic cyclic rings comprising one or more basic nitrogen atoms. Many plants and animals produce them as secondary metabolites. Despite this diversity, plant-derived chemical candidates have been used to screen, identify, and uncover pharmacologically relevant alkaloids for disease treatment (26). Alkaloids are present in the leaves, seeds, stems, roots, and leaves of higher plants in the *Loganiaceae Papaveraceae, Ranunculaceae, Solanaceae, Amaryllidaceae, Menispermaceae, and Solanaceae*, families. They are mostly generated from amino acid precursors (27). Their extensive range of pharmacological actions such as antibacterial, anticancer, antioxidant, anti-inflammatory, anticholinergic, antiviral, neurological, and cardiovascular stems from their structural and chemical diversity (28, 29). One of the most diverse and intensively studied natural product classes is alkaloids. Alkaloids are natural bioactive compounds that contribute significantly to the development of anticancer drugs. About 60% of anticancer medicines come from plant bioactive compounds. The tubulin-binding alkaloids vincristine and vinblastine are extensively used to treat hematological and lymphatic neoplasms due to their ability to disrupt microtubule dynamics and inhibit mitotic progression (30). Colchicine, alone or with taxane and Vinca alkaloids, exhibits considerable chemotherapeutic potential through its microtubule disrupting activity (31). Berberine, an isoquinoline alkaloid inhibits cell growth via interacting with miRNAs and inhibiting telomerase (32, 33).

## 4 Modulated miRNA expression and cancer progression

miRNAs are a class of endogenous, small non-coding RNA molecules ranging from average 18–22 bps in length. To date, approximately 2654 mature human miRNAs have been reported (34). These miRNAs act as critical regulator of gene expression at post-transcriptional level, influencing a wide range of physiological and cellular processes. miRNAs associate with the target mRNA in the cytoplasm, leading to its degradation or translation repression (35). Specifically, downregulation of a miRNA increases protein expression and vice versa. In contrast, miRNA overexpression decreases target protein(s) expression. miRNAs repress mRNA translation by binding to sites located within 3′ UTRs, 5′ UTRs and coding sequences. Certain miRNAs can also interact with promoters region of genes and may contribute to activate gene transcription (36). Although miRNAs are primarily intracellular, lipid coated exosomes also release certain miRNAs from cells (37). Stable in circulation and secured from endogenic RNAse activity, circulating exosomal miRNAs may signify disease biomarkers (38). miRNAs contribute to alter cancer cells. miRNAs target genes intricated in tumor formation and cell-cycle inhibition to suppress or activate tumors. Calin et al. discovered miRNA dysregulation in chronic lymphocytic leukemia 20 years ago. Most B-cell CLL is linked to the loss at chromosome 13q14, yet researchers have found no tumor-suppressing genes there. Calin et al. found that genomic regions encoding miR-15 and miR-16 is located within a ~30 kb deleted segment frequently observed in CLL, suggesting a tumor suppressive role of these miRNAs (39). Subsequent investigations revealed that miRNAs were found to regulate all cancer hallmarks. miRNAs may either suppress cancer progression by functioning as tumor suppressors or promote carcinogenesis by acting as oncogenic miRNAs (oncomiRs).

The miR-17–92 cluster is situated within intron 3 of the C13orf25 gene at 13q31.3 and generating six mature miRNAs (miR-17, -18a, -19a, -20a, -19b, and -92a). This cluster was aberrantly elevated in lung cancer with instances of genomic amplification (40). The cluster’s miR-92a targets Bcl-2 expression and is upregulated in colon cancer (41). miR-21, another extensively reported oncomiR, is frequently upregulated in various types of carcinomas. In prostate, breast, ovarian, and gastrointestinal carcinomas, overexpression of miR-21 levels has been reported (42–45). miR-21 is involved in tumor start and progression in colorectal and gastric cancer, and its high level may be a prognostic marker (46). The firstly identified miRNA, Let-7, suppresses oncogenes Ras and c-Myc (47). Lung, breast, stomach, colon, prostate, and other malignancies have been linked to let-7 family members’ tumor-suppressing role. In non-small cell lung cancer, expression of let-7 is downregulated. Ectopic expression studies in mice produce lung cancer cell cycle arrest and death via Ras regulation. Let-7’s significance in lung cancer tumor reduction was confirmed (48).

Since miR-29 suppresses tumor growth, its downregulation has been linked to invasion and metastasis of tumor various types of malignancies (49). Low miR-29 expression in osteosarcoma increases cell proliferation and inhibits apoptosis. STAT3 which is aberrantly upregulated in osteosarcoma, has been identified as a direct gene target of miR-29 (50). Another tumor-suppressing miRNA, miR-34, is frequently downregulated across multiple malignancies. Restoring miR-34 levels in pancreatic cancer decreased Bcl-2 and Notch 1/2 expression and hindered cell proliferation and invasion (51). Since their discovery, miRNAs have emerged as promising biomarkers and shown promise for cancer detection, prognosis, and treatment. Diverse tumor types have diverse miRNA profiles, which can be used as phenotypic signatures for cancer diagnosis, prognosis, and treatment. If miRNA profiles can effectively predict malignancies, this approach potentially solve numerous diagnostic problems (52).

## 5 Alkaloids as miRNA modulators in different types of cancer

Alkaloids are naturally occurring, nitrogenous organic molecules with alkaline properties that are mostly extracted from plants. These compounds generally possess intricate ring structures that include nitrogen within the rings (53). Alkaloids have significant biological activity and are essential active constituents of traditional Chinese herbal remedies (54). Numerous studies revealed that various alkaloids, including berberine and matrine, exhibit significant anticancer properties (55, 56). Recent research shows that these natural compounds target many miRNAs and their downstream effectors implicated in cancer growth. Thus, we have delineated the miRNA-modulating functions of several alkaloids across different cancer types (**Table 1**).

**Table 1:** Natural alkaloids as miRNA modulators and their associated anticancer mechanism in different types of carcinomas.

Alkaloid	Type	Cancer type	Target miRNA	Anticancer effects	Dose	Reference
Berberine	Benzylisoquinoline alkaloid	Multiple myeloma (RPMI-8266 and U266 cells)	Downregulated miR-17∼92, miR-99a∼125b, and miR-106∼25	Apoptotic induction and G2 cell cycle arrest by downregulating TP53, Erb and MAPK expression	75 μM	(57)
		Multiple myeloma (U266 cells)	Downregulated miR-21	ROS generation and apoptotic induction by suppressing NF-kB translocation	80-160 μmol/L	(58)
		Multiple myeloma (RPMI-8266 and U266 cells)	Downregulated miR-21	Apoptotic induction, G2 cell cycle arrest and colony inhibition through IL6/STAT3 downregulation	75 and 120 μM	(59)
		Endometrial cancer (AN3-CA and HEC-1-A cells; HEC-1-A cells implanted xenograft mice model; tissue samples)	Upregulated miR-101	Inhibited tumor growth by COX-2/PGE2 signaling pathway	25 and 50 μM	(60)
		Endometrial cancer (ishikawa and HEC-1A cells)	Upregulated miR-377-3p	Inhibited cell proliferation and metastasis by COX-2 mediated pathway	10-80 μM	(61)
		B-chronic lymphocytic leukemia (Patients sample)	Downregulated miR-21	Apoptotic induction by downregulating Bcl-2 and ROR1 expression	25 μM	(62)
		Chronic lymphocytic leukemia (Patients sample)	Downregulated miR-155	Antitumor activity by downregulating CD69, Ki67, and upregulating cleaved PARP1 activity	25 μM	(63)
		Breast cancer (MCF-7 cells)	Upregulated miR-21-3p	Antitumor activity by downregulating CYP1A1 activity	10 μM	(64)
		Breast cancer (MDA-MB-231 and MCF-7 cells)	Downregulated miR-214-3p	Inhibited cell growth, cell cycle arrest (G2/M), and apoptosis induction by downregulating secretin expression	50 μM	(65)
		Breast cancer (4T1 and MDA-MB-231 cells; 4T1 cells induced xenograft mice model)	Upregulated miR-let-7c and miR-34a-5p	Suppressed tumor growth and breast cancer stem cells development by inhibiting IL-6 signaling.	75 μM	(66)
		Ovarian cancer (SKOV3 and 3AO cells)	Upregulated miR-145	Inhibited cell proliferation, invasion and migration through MMP16 downregulation	78.52 µM and125.8 µM	(67)
		Ovarian cancer (A2780/DDP cells)	Downregulated miR-93	Augmented cisplatin induced apoptosis and cell cycle arrest through PTEN/AKT pathway	5-20 µM	(68)
		Ovarian cancer (SKOV3 and OVCAR3 cells)	Downregulated miR-21	Augmented cisplatin induced apoptosis by upregulating PDCD4 levels.	1-100 µM	(69)
		Bladder cancer (T24, 5637 cells; T24 cells implanted xenograft mice model)	Upregulated miR-17-5p	Inhibited cell proliferation, invasion, migration and induced apoptosis through JAK1/STAT3 signaling pathway	40 and 60 μM	(70)
		Hepatocellular carcinoma (HepG2 cells)	Upregulated miR-21-3p	Repressed cell proliferation and induced apoptosis by decreasing the expression of MAT2A and MAT2B	40 µM	(71)
		Hepatocellular carcinoma (HepG2 cells)	Upregulated miR-23a	Antitumor activity by upregulating p53 activity	50 and 100 μM	(72)
		Gastric cancer (SGC-7901/DDP and BGC-823/DDP)	Upregulated miR-203	Enhanced cisplatin sensitivity by inducing caspase-dependent apoptosis	20-40 μM	(73)
Coptisine	Isoquinoline	Hepatocellular carcinoma (HepG2 and Huh7 cells; HepG2 cells transfected xenograft mice)	Upregulated miR-122	Suppressed cell proliferation, migration and apoptosis induction	25 μg/mL	(74)
		Lung Cancer (A549 and Calu-1 cells)	Upregulated miR-128-3p	Antitumor activity by suppressing TGF-β and VEGFC signaling cascade	20-40 μM	(75)
Evodiamine	Indole-quinazoline	Ovarian cancer (SKOV3, ES- 2, and OVCAR- 3)	Upregulated miR-152-3p	Inhibited cell growth, cell cycle arrest (G2/M), and caspase mediated apoptosis induction by downregulating CDK19 expression	2.5-10 μM	(76)
		Colorectal cancer (Tissue samples)	Downregulated miR-429	Tumor suppression by restoring epithelial proteins E-cadherin and Par3	3-12 μM	(77)
Indole-3-carbinol	Indole	Hepatocellular carcinoma (SK-Hep-1 and SNU-449 cells; SK-Hep-1 cells implanted xenograft mice model)	Downregulated miR-21, miR-221, miR-222	Antitumor activity by suppressing PTEN/AKT signaling cascade	50-200 μM	(78)
		Hepatocellular carcinoma (HepG2 cells)	Upregulated miR-34a	Growth inhibition, suppressing aerobic glycolysis and stabilization of p53	200 μM	(79)
		Pancreatic cancer (Panc-1 cells)	Downregulated miR-21	Enhanced gemcitabine sensitivity and apoptosis induction by upregulating PDCD4 level	50, 100, 200 μM	(80)
		Breast cancer (MCF-7 and T47D cells)	Upregulated miR-34a	Inhibited cell proliferation by p53 dependent and independent pathways	200 μM	(81)
Matrine	Tetracyclic quinolizidine	Lung cancer (A549 cells)	Upregulated miR-126	Inhibited cell growth, cell cycle arrest (G1), and apoptosis induction by downregulating VEGF expression	0.2-1.0 mg/ml	(82)
		Papillary thyroid cancer (TPC-1, BCPAP, and K1 cells; TPC-1 transfected xenograft mice model)	Downregulated miR-182-5p	Antitumor effects by inducing caspase-mediated apoptosis and downregulating Bcl-2 level	0.25, 0.5, 1 mg/ml	(83)
		Gastric cancer (SGC-7901 and MKN-45 cells)	Downregulated miR-93-5p	Inhibited cell proliferation, invasion, and migration through upregulation of AHNAK	1–2 mg/mL	(84)
		Colorectal cancer (HT29 and DLD1 cells; patient tissue samples);	Downregulated miR-10b-5p	Suppressed cell growth, migration, invasion, and apoptotic induction by PTEN pathway	0.4, 0.8 mg/ml	(85)
		Colon cancer (SW480 and SW620 cells)	Upregulated miR-22	Induced G0/G1 cell cycle arrest, and apoptosis through suppressing Wnt/β-catenin and MEK/ERK pathways	0.25, 0.5, 0.75, 1, and 1.25 mM	(86)
		Breast cancer (MCF-7 cells)	Downregulated miR-21	Growth inhibition, G1/S cell cycle arrest, and apoptosis induction through PTEN/Akt pathway	1–2 mg/mL	(87)
		Melanoma (A375 and SK-MEL-2 cells)	Downregulated miR-19b-3p	Suppressed cell growth, migration, invasion, and apoptotic induction by PTEN pathway	250 and 500 μg/mL	(88)
		Acute myeloid leukemia (THP-1 and HL-60 cells)	Upregulated miR-495-3p and miR-543	Suppressed cell growth, glycolysis, and induced apoptosis through suppressing Wnt/β-catenin pathway	1-2 mM	(89)
Neferine	Bisbenzylisoquinoline	Breast cancer (MDA-MB-231 cells)	Downregulated miR-374a	Suppressed cell growth, migration, invasion, and apoptotic induction via regulating FGFR-2 mediated PI3K/AKT and MEK/ERK pathway	8 μM	(90)
		Nasopharyngeal carcinoma (5-8F/Taxol and CNE-1/Taxol cells; 5-8F/Taxol cells transfected xenograft mice model)	Downregulated miR-130b-5p	Enhanced taxol sensitivity by regulating EMT process.	30 μM	(91)
Piperlongumine	Amide	Non-small-cell lung cancer (A549, H520, SPC-A-1, and H1299 cells; A549 cells transfected xenograft mice model)	Upregulated miR-34b-3p	Suppressed cell growth, and apoptotic induction by regulating TGFBR1 pathway	10 μM	(92)
		Osteosarcoma (U2OS and MG63 cells)	Downregulated miR-30d-5p	Suppressed cell growth, migration, invasion, EMT, and apoptotic induction via modulating SOCS3 mediated JAK2/STAT3 pathway	2.5, 5 and 10 μM	(93)
		Pancreatic cancer (Panc1 cells)	Downregulated miR-27a, miR-17, miR-20a	ROS mediated c-Myc via epigenetic pathway	5 mmol/L	(94)
Sanguinarine	Benzylisoquinoline	Gastric cancer (BGC-823 cells; BGC-823 cells transfected xenograft mice model)	Downregulated miR-96-5p, miR-29c-3p	Inhibited cell proliferation and stimulation of MAPK/JNK signaling	2 and 4 μM	(95)
		Hepatocellular carcinoma (L02 cells and HepG2 cells)	Upregulated miR-497-5p, miR-29c-3p	Inhibited cell proliferation, invasion, and induced apoptosis by downregulating CDK4 expression	1 and 2 μM	(96)
		Hepatocellular carcinoma (HepG2, Huh7 and Hep3B cells)	Upregulated miR-16-2	Attenuated tumor growth, cell cycle progression, apoptotic induction by upregulating p53 expression	8 μM	(97)
Sinomenine	Morphinan derivative	Lung cancer (A549, and H1299 cells)	Downregulated miR-21	Inhibited cell proliferation, invasion, and EMT by downregulating MMPs expression	0.05, 0.1 and 0.2 mM	(98)
		Breast cancer (MDA-MB-231 cells)	Upregulated miR-340-5p	Inhibited hypoxia induced vasculogenic mimicry and EMT through downregulation of SIAH2 and HIF-1α	0.75 mM	(99)
		Oral squamous cell carcinoma (Tissue samples; OECM-1, CAL33, SCC-15, HSC-3, and HSC-4 cells; CAL33 5-fluorouracil resistant cells transfected xenograft mice model)	Upregulated miR-140-5p	Enhanced 5-fluorouracil sensitivity and suppressed anaerobic glycolysis by targeting PDK1	100, 200 or 400 μM	(100)
Sophocarpine	Quinolizidine	Head and Neck Cancer (UM-SCC-22B and UM-SCC-47 cells; UM-SCC-22B cells transfected xenograft mice model)	Downregulated miR-21	Inhibited cell proliferation, invasion, migration and EMT by PTEN/p38MAPK signaling pathway	1, 2 and 4 μM	(101)
		Glioblastoma (LN229 and SF539 cells)	Downregulated miR-21	Suppressed cell growth, and stimulated apoptosis induction, EMT by increasing PTEN expression and suppressing PI3K/AKT pathway	0.05–1 mM	(102)
Nitidine Chloride	Benzophenanthridine	Chronic myeloid leukemia (K562 and K562/G01 cells; tissue samples)	Downregulated miR-17, miR-20a	Stimulated erythroid differentiation and apoptosis through downregulation of c-Myc	4 and 8 μM	(103)
		Hepatocellular carcinoma (Clinical tissue samples)	Upregulated miR-125b-2-3p	Antitumor effects by targeting PRKCA	–	(104)
Etoposide	Epipodophyllotoxin	Breast cancer (MCF-7 cells)	Upregulated miR-205-5p	Suppressed cell growth, and migration and EMT through downregulation of ERBB4 expression	50 and 100 μM	(105)
		Hepatocellular carcinoma (HepG2 cells)	Upregulated miR-23a	Antitumor effects by downregulating TOP1 expression	20 μM	(106)
Tetrandrine	Bisbenzylisoquinoline	Cervical cancer (HeLa and SiHa cells)	Upregulated miR-638	Inhibited cell proliferation, invasion, and migration through NCAPG2 modulation.	30 μM	(107)

### 5.1 Berberine

Berberine has been shown to exert anticancer activity largely through the modulation of oncomiRs and their associated signaling networks. Transcriptomic analysis indicated that berberine significantly downregulated several oncomiRs clusters, including miR-99a~125b, miR-17~92 and miR-106~25 (57), as well as the key oncmiR miR-21, leading to altered expression of critical cancer-related genes such as RAC1, NF-κB, MYC, JUN, and CCND1. Mechanistically, berberine interferes with major regulatory pathways, including TP53, MAPK, and Erb signaling and inhibit NF-κB activation through Set9-mediated methylation of the RelA subunit, thereby reducing miR21 transcription (58). In addition, suppression of IL-6/STAT3 axis further contributes to miR-21 downregulation and subsequent upregulation of tits tumor-suppressive target PDCD4 (59). These molecular events further promote ROS generation, G2/M cell cycle arrest, apoptosis and inhibition of cell growth and colony formation in multiple myeloma cells following berberine exposure.

Wang et al. shown that berberine enhances miR-101 transcription through AP-1 activation, thereby inhibiting COX-2 expression and downstream PEG2 signaling in both *in vitro* and *in vivo* endometrial cancer models (60). Another work conducted by Liang et al. found that berberine exerts its anticancer effects in endometrial cancer through the downregulation of circ_ZNF608 and the upregulation of miR-377-3p levels, resulting in suppression of COX-2 expression (61).

Chronic lymphocytic leukemia (CLL) is often linked to altered expression of miRNAs, which is associated with poor prognosis. Experimental studies using peripheral blood mononuclear cells from CLL patients have demonstrated that berberine treatment reduces the expression of miR-21 and miR-155, along with a decreased levels of antiapoptotic protein Bcl-2, ROR1, CD69, and Ki67. This modification elevates the Bax/Bcl2 ratio and cleaved PARP activity, signifying augmented apoptosis in leukemic cells (62, 63).

In preclinical breast cancer models, berberine consistently regulates miRNA expression to control tumor-related cellular pathways. A study by Lo et al. examined the effects of berberine on AhR signaling and CYP1 enzyme level in MCF-7 breast cancer cells. Berberine markedly increased CYP1-related mRNA levels; however, it did not augment the corresponding protein activity because miR-21-3p was overexpressed, inhibiting protein expression (64). Another study by Zhu et al. investigated the anticancer effects of berberine on MCF-7 and MDA-MB-231 cells by targeting miR-214-3p. Results showed that berberine upregulated miR-214-3p levels, which led to reduced cell growth, migration, and invasion, and stimulated cell cycle arrest and apoptosis (65). Ibrahim et al. investigated effect of berberine on breast cancer stem cells, inflammation, and behavior. Berberine inhibited stem cell proliferation and mammosphere formation. It further upregulated tumor suppressive miRNAs, miRNA-let-7c and miR-34a, and reduced serum IL-6 levels *in vivo*, suggesting anti-inflammatory and epigenetic properties (66).

In ovarian cancer, berberine demonstrated significant potential as a regulator of miRNAs, affecting a variety of pathways that are involved in the progression of the tumor and the response to chemotherapy. Multiple studies indicated that its anticancer efficacy is closely linked to its ability to regulate specific miRNAs and their downstream targets. Berberine has been shown to enhance miR-145 expression and inhibit MMP16, a protein linked to cancer cell proliferation, migration, and invasion. Berberine significantly restricts essential processes necessary for tumor growth and metastatic progression via the miR-145/MMP16 axis (67). In addition to its direct anticancer properties, berberine also enhances chemosensitivity in ovarian cancer cells. In cisplatin-resistant ovarian cancer models, berberine reduces the expression of drug resistance-associated miR-93. This downregulation promotes apoptosis and reduces cell survival by inhibiting downstream AKT signaling and restoring PTEN activity, a crucial tumor suppressor. These results underscore the significance of the miR-93/PTEN/AKT pathway in facilitating the chemosensitising effects of berberine (68). Likewise, berberine has been shown to target miR-21, a miRNA commonly linked to chemotherapy resistance. By inhibiting miR-21, berberine enhances PDCD4 expression, a tumors suppressor implicated in apoptosis regulation. This modification enhances the efficacy of cisplatin and increases cancer cell mortality (69).

In bladder cancer, Xie et al. demonstrate that berberine exerts potent anti-tumor effects by upregulating miR-17-5p and regulating oncogenic signaling pathways. This miRNA directly targets the 3’UTRs of JAK1 and STAT3, leading to their suppression. As a results, berberine effectively inhibits the JAK1/STAT3 signaling cascade, which plays a central role in tumor progression, metastasis, and chemoresistance. Berberine significantly reduced cell growth of bladder cancer cells, *in vitro* and *in vivo*, with comparatively low toxicity against urothelial cells (70).

Berberine has also attracted as a miRNA networks modulator in gastrointestinal cancers, affecting their tumor proliferation, apoptosis, and chemotherapy responsiveness through multiple molecular mechanisms. In hepatocellular carcinoma, berberine has been shown to alter multiple tumor-suppressive miRNAs. A study by Lo et al. showed that berberine increases miR-21-3p levels, which directly target MAT2A and MAT2B, essential enzymes in cellular methylation metabolism. This targeting resulted in elevated intracellular S-adenosylmethionine levels and establishes conditions that hinder tumor cell proliferation in hepatocellular carcinoma (71).

In another study, berberine enhances miR-23a expression at various stages of its maturation, and this overexpression is tightly associated with p53 function. Functionally, miR-23a facilitates the transcriptional activation of p53 downstream targets, including p21 and GADD45α, hence increasing tumor-suppressive signaling. Moreover, miR-23a targets Nek6, downregulating its expression and facilitating cell cycle arrest at the G2/M phase. The suppression of miR-23a diminishes these effects, highlighting its pivotal role in promoting berberine-induced growth inhibition and cellular apoptosis (72).

In addition to liver cancer, berberine significantly enhances chemosensitivity in gastric cancer by increasing drug sensitivity and apoptosis-induction via caspase-dependent pathways. This action is linked to the overexpression of miR-203, which directly targets the anti-apoptotic protein Bcl-w (73). These data collectively underscore berberine’s versatility as a regulator of miRNA expression in gastro intestinal malignancies.

**Figure 1:**
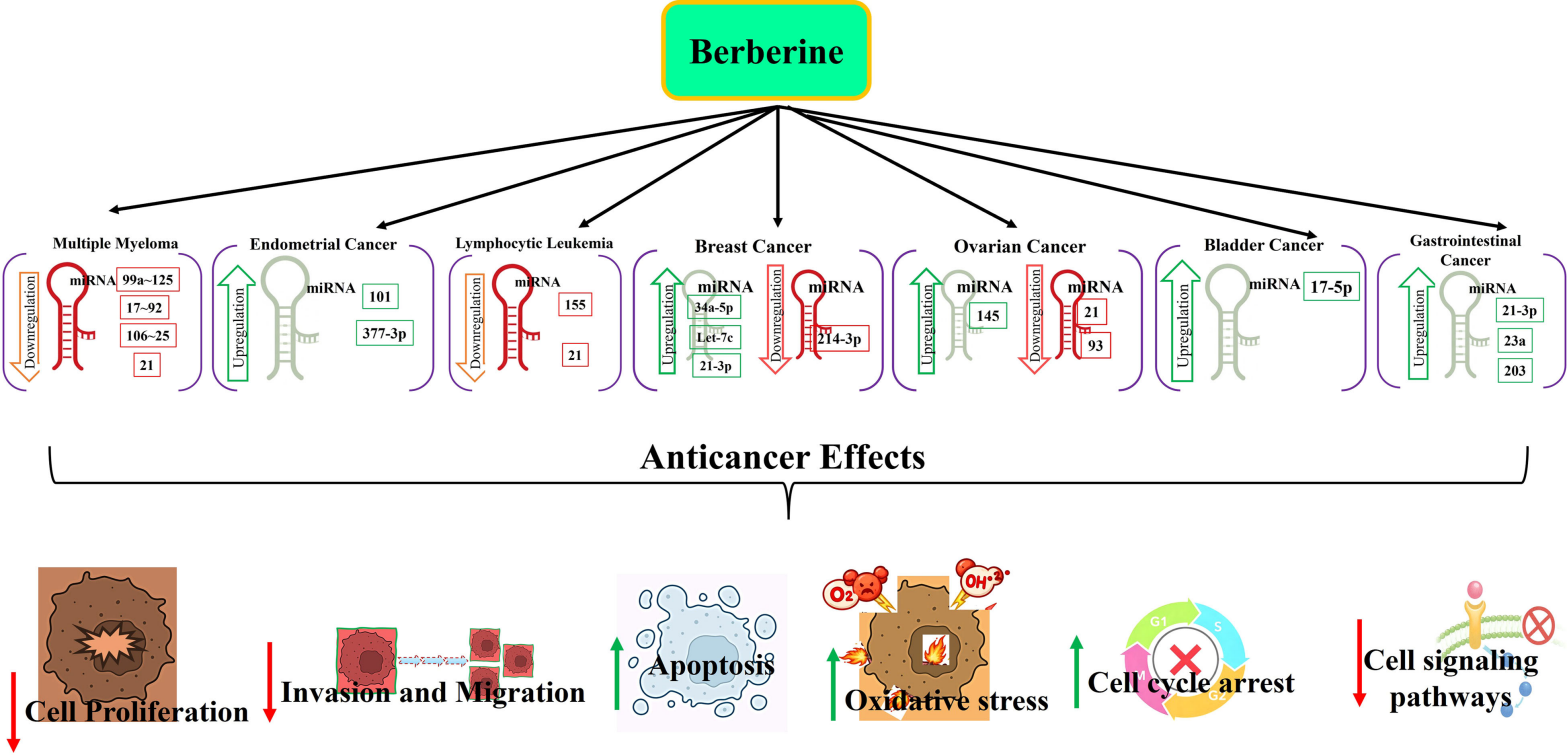
Anticancer effects of berberine by targeting miRNA across various cancer types. Bereberine modulates the expression of oncogenic and tumor suppressive miRNAs, leading to inhibition of various oncogenic processes including cell proliferation, invasion and migration. Additionally, berberine induces cell cycle arrest, ROS mediated apoptosis and modulates key cell signaling pathways associated with tumor progression.

Collectively, berberine modulates the expression of numerous oncogenic and tumor-suppressive miRNAs across various cancer types, thus influencing critical processes such as cancer cell proliferation, invasion, migration, and apoptosis (**Figure 1**).

### 5.2 Coptisine

Coptisine is an isquinoline alkaloid mainly extracted from *Coptis chinensis* and actively used in Chinese medication for treating various ailments (108, 109). It has gained significant attention due to its significant anticancer effects, including suppression of cell proliferation, angiogenesis, and metastasis (110, 111). Coptisine inhibits tumor growth by regulating various oncogenic pathways, including NF-κB, STAT3, PI3K/AKT, and MAPK/mTOR signaling (112–114). Recent evidence suggested that coptisine also influences ROS generation and mitochondrial pathway of apoptosis in cancer cells (115, 116).

**Figure 2:**
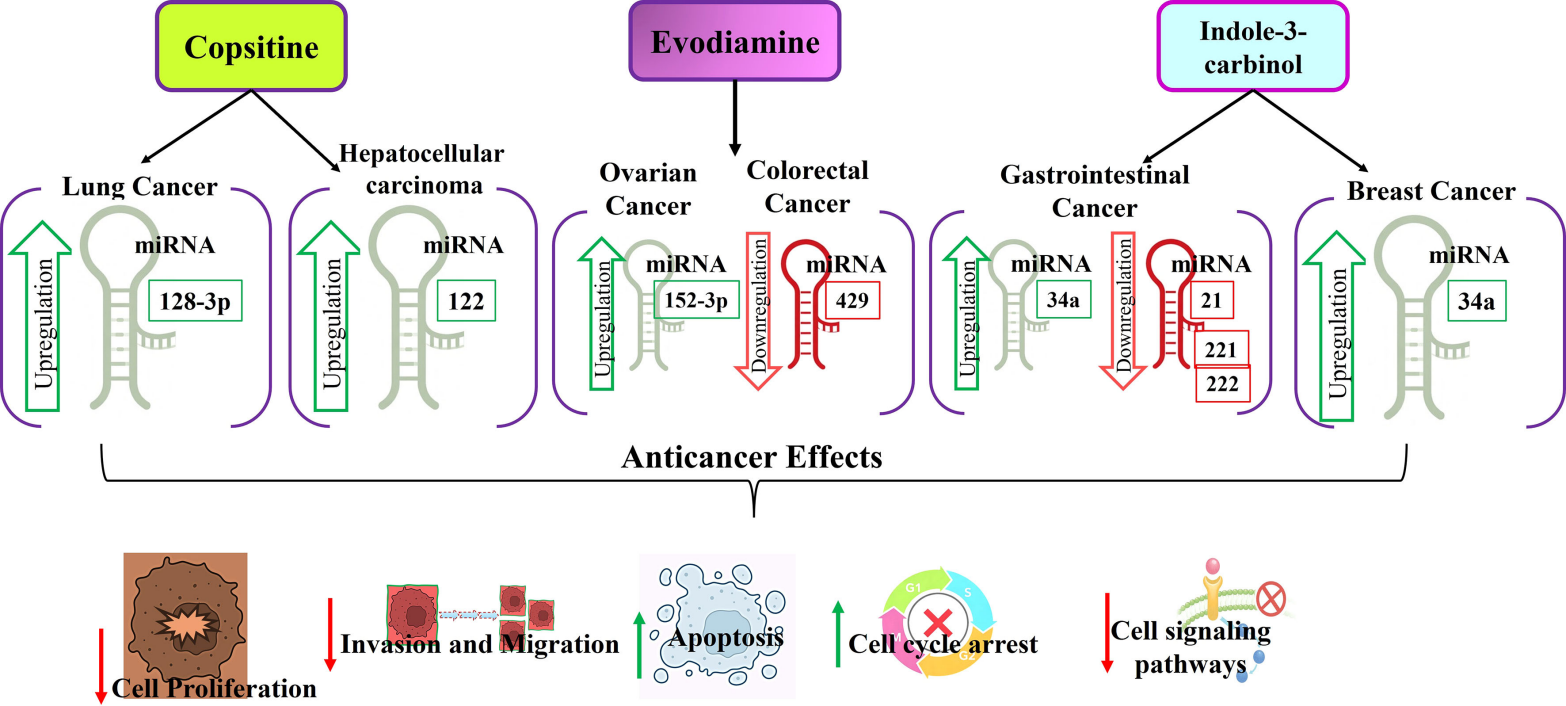
Anticancer effects of copsitine, evodiamine and indole-3-carbinol by targeting miRNA across various cancer types. These alkaloids modulate the expression of oncogenic and tumor suppressive miRNAs, leading to inhibition of various oncogenic processes including cell proliferation, invasion and migration. Additionally, these alkaloids induce cell cycle arrest, apoptosis and modulates key cell signaling pathways associated with tumor progression.

Coptisine has shown considerable anticancer efficacy in hepatocellular carcinoma by increasing the expression of tumor suppressive miR-122, both *in vitro* and *in vivo*. This modification resulted in decreased cell proliferation and migration of HepG2 cells, as well as increased apoptosis. Additionally, it effectively inhibited tumor growth in nude mice in a manner similar to sorafenib (74). Another study by Gu et al. reports that coptisine exhibits substantial anticancer properties in lung cancer A549 and Calu-1 cells, inhibiting cell growth and invasion, and inducing apoptosis. Coptisine significantly upregulated miRNA-128-3p levels while downregulating MSTO2P expression, highlighting a negative regulatory interaction between the two (75). The findings underscore coptisine as a potential therapeutic agent targeting miRNA mediated pathways in cancers (**Figure 2**).

### 5.3 Evodiamine

Evodiamine is an indole-quinazoline alkaloid extracted from the fruit of *Evodiae fructus*. Increasing evidence indicated that evodiamine displays a wide range of biological actions, including its efficacy in treating cancer, autoimmune, and inflammatory disorders (117–120). The antitumor efficacy of evodiamine has garnered the attention of researchers. Evodiamine functions as a dual catalytic inhibitor of the nuclear enzymes topoisomerase I and II, which are essential targets for anticancer drugs (121). Furthermore, mounting evidence indicates that evodiamine demonstrates promising anticancer potential both *in vitro* and *in vivo* (122–124).

Evodiamine demonstrates significant anticancer efficacy in ovarian cancer cells by inhibiting proliferation and triggering G2/M phase cell cycle arrest, thereby restricting tumor development. It facilitates apoptosis by modulating essential apoptotic proteins, specifically by downregulating BCl-2 level and upregulating Bax and cleaved caspase-3 levels, indicating the activation of intrinsic apoptotic pathway. Evodiamine upregulates miR-152-3p at the molecular level, which subsequently inhibits oncogenic targets including CDK19, and is negatively controlled by the lncRNA NEAT1. Evodiamine disrupts the NEAT1/miR-152-3p/CDK19 regulatory axis, hence interfering with essential signaling pathways that regulate cell survival, proliferation, and apoptosis (76). A study by Liu et al. illustrated the anticancer efficacy of evodiamine in colorectal cancer by altering miRNA expression, namely by the downregulation of miR-429. Increased miR-429 in tumor tissues correlated with diminished expression of epithelial markers, including E-cadherin and Par3, essential for preserving cell adhesion and polarity. Evodiamine may restore epithelial structure and limit tumor progression by inhibiting miR-429, suggesting its potential significance in correlating the malignant traits of colorectal cancer through miRNA-mediated modulation (77). These multitargeted modulation underscores its promise as an efficacious therapeutic agent in cancer treatment (**Figure 2**).

### 5.4 Indole-3-carbinol

Indole-3-carbinol is a natural phytochemical alkaloid prevalent in cruciferous vegetables. It exhibits significant anticancer potential due to its ability to disrupt multiple carcinogenic signaling pathways (125, 126). Most studies examining the indole-3-carbinol;s potential to affect cancer incidence have concentrated on its effects on estrogen metabolism (127). Concurrently, mounting data indicated that indole-3-carbinol attenuates cell growth and stimulates apoptosis by affecting many signaling pathways related to cell survival, cell cycle regulation, and apoptosis (128–130).

Indole-3-carbinol exhibits significant anticancer properties in hepatocellular carcinoma by targeting miRNA-mediated oncogenic signaling pathways. It markedly downregulates oncomiRs, miRNA-21 and miR-221/222, which are known to inhibit tumor suppressor genes. PTEN expression is subsequently restored, leading to repression of the AKT pathway, an essential driver of cell survival, proliferation, and migration. Indole-3-carbinol inhibits tumor cell proliferation, reduces wound healing activity, and stimulates apoptosis in preclinical models of hepatocellular carcinoma. It is also reported that indole-3-carbinol enhances the susceptibility of cancer cells to treatment by reducing miR-21-mediated drug resistance, suggesting its potential to overcome therapeutic resistance in hepatocellular carcinoma (78). Another hepatocellular carcinoma study of indole-3-carbinol demonstrated that indole-3-carbinol stabilizes p53 by inhibiting MDM2-mediated degradation, thereby increasing p53 activity. Furthermore, indole-3-carbinol exposure stimulates miR-34a, which targets and downregulates LDHA, a crucial enzyme in aerobic glycolysis, therefore impairing cancer cell metabolism in HepG2 cells (79).

Paik et al. demonstrated that indole-3-carbinol enhanced anticancer effects in pancreatic cancer by downregulating oncomiRs-21. This miRNA inhibition stimulates PDCD4 expression, leading to enhanced apoptosis and increased gemcitabine sensitivity in Panc-1 cells. The restoration of miR-21 significantly counteracts these effects, validating that indole-3-carbinol-induced chemosensitization functions via miR-21 regulation (80). Hargraves et al. reported that indole-3-carbinol exhibits antiproliferative effects in breast carcinoma cells by triggering cell cycle arrest via p53-dependent overexpression of miR-34a. Increased levels of miR-34a block its target gene CDK4, thereby obstructing cell cycle progression (81). Altogether, indole-3-carbinol demonstrated a multifaceted anticancer effect and enhances chemotherapy efficacy by altering miRNA expression and disrupting critical survival pathways across several cancer types (**Figure 2**).

### 5.5 Matrine

Matrine, an alkaloid derived from the root of Sophora flavescens, demonstrates multiple therapeutic properties, including anti-inflammatory, anticancer, and antipyretic activities (131). Among the numerous pharmacological properties, anticancer activity of matrine has gained a lot in recent years. Matrine demonstrated its anticancer efficacy by numerous mechanisms including suppressing tumor growth, enhances chemosensitivity, and reducing toxicity towards normal cells (132–135).

An et al., demonstrated that matrine induces anticancer effects in lung cancer cells, partially by upregulating miR-126 expression. Treatment with matrine inhibited A549 cells proliferation by inducing cellular damage and cell cycle arrest at G0/G1 phase. Upregulation of miR-126 is associated with decreased expression of VEGF following exposure to low concentration of matrine (82). Research by Fu et al. demonstrates that matrine exhibits anticancer properties in papillary thyroid carcinoma by downregulating the oncogenic miR-182-5p. Furthermore, suppressed activity of miR-182-5p led to Bcl-2 downregulation and increased caspase-3 activation, resulting in increased apoptosis. Matrine additionally suppresses tumor proliferation both *in vitro* and *in vivo*. The restoration of miR-182-5p mitigates these effects, thereby affirming its pivotal function in facilitating the action of matrine (83).

Matrine inhibits gastric cancer cell growth and migration by markedly by reducing miR-93-5p levels. Moreover, miR-93-5p targets the AHANK gene, and the inhibition of this miRNA by matrine results in elevated AHNAK expression (84).

Matrine demonstrates anticancer properties in colorectal cancer by modulating miRNAs in distinct ways. A study demonstrates that matrine inhibits tumor cell proliferation, motility, and invasion, and induces apoptosis by downregulating miR-10b-5p. Furthermore, matrine treatment upregulated PTEN expression in these colon cancer cells. This miRNA-mediated mechanism facilitates diminished tumor proliferation both *in vitro* and *in vivo* (85). In the second study, matrine elevates miR-22, leading to increased apoptosis and G0/G1 cell cycle arrest. This result is associated with the suppression of critical oncogenic signaling pathways including Wnt/β-catenin and MEK/ERK, by targeting genes such as ERBB3 and MECOM (86). In breast cancer cells, matrine suppressed cell proliferation, arrests cell cycle at the G1/S phase, inducing apoptosis by downregulating miR-21, an oncomiRs. Decreased miR-21 levels lead to PTEN overexpression and subsequent suppression of Akt activity. Consequently, matrine alters the expression of pro-apoptotic and cell cycle regulating proteins, including Bad, p21, and p27, hence enhancing matrine’s anticancer efficacy (87). Matrine inhibits melanoma cell proliferation and invasiveness while enhancing apoptosis by downregulating miR-19b-3p expression. This reduction leads to overexpression of PTEN, a tumor suppressor gene directly regulated by miR-19b-3p. The reversal of these effects following PTEN silencing substantiates that matrine demonstrates its anticancer efficacy through the miR-19b-3p/PTEN signaling (88).

**Figure 3:**
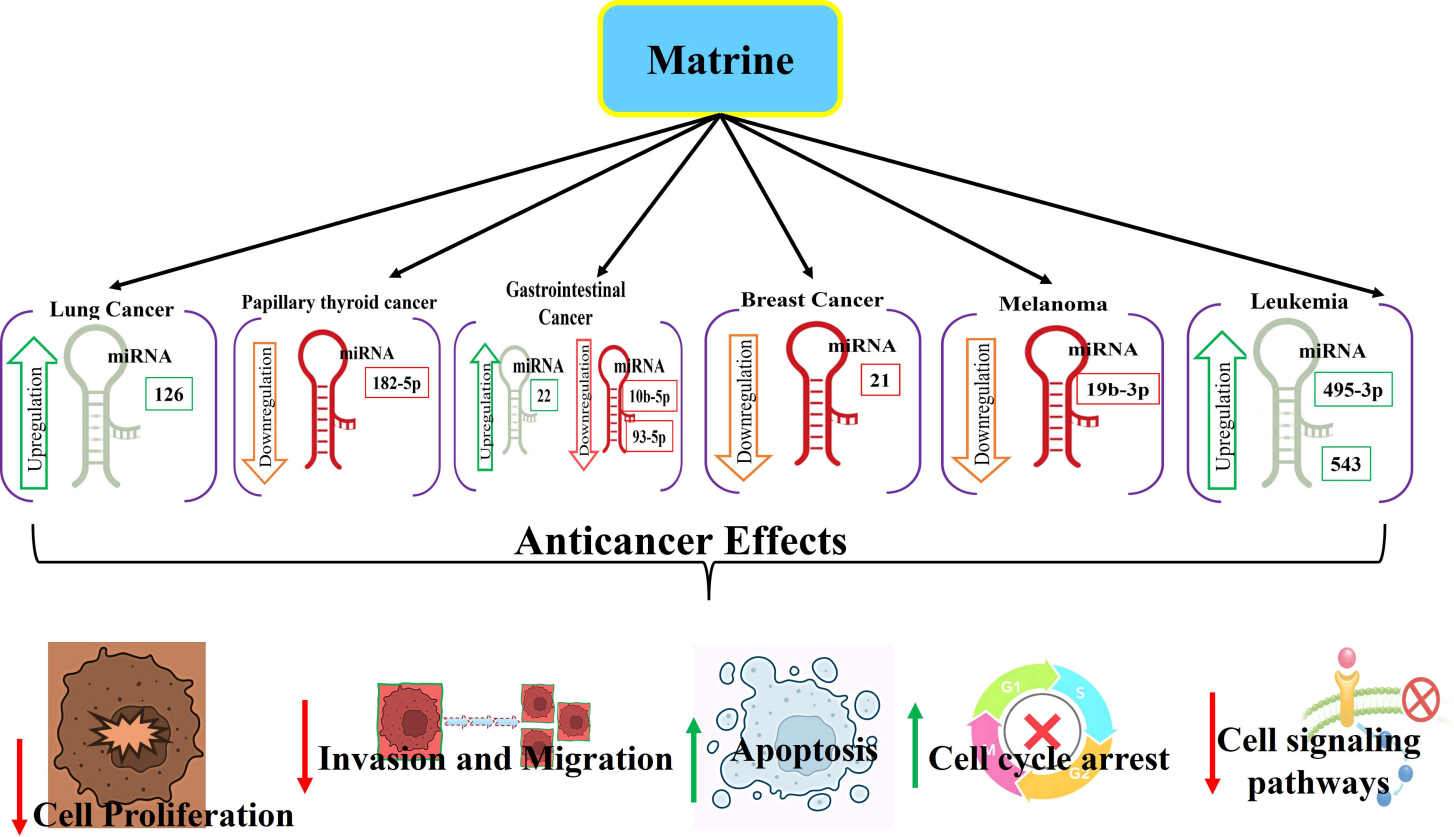
Anticancer effects of matrine by targeting miRNA across various cancer types. Matine modulates the expression of oncogenic and tumor suppressive miRNAs, leading to inhibition of various oncogenic processes including cell proliferation, invasion and migration. Additionally, matrine induces cell cycle arrest, apoptosis and modulates key cell signaling pathways associated with tumor progression.

Lei et al. established that matrine inhibits the growth of acute myeloid leukemia by restraining cell proliferation and glycolysis, while promoting apoptosis and cell cycle arrest. The effects are mediated by the control of the miR-495-3p/miR-543 axis, as these miRNAs directly target the tumor growth regulator, PDK1. Matrine modulates this pathway to diminish PDK1 expression, thereby suppressing the Wnt/β-catenin pathway (89). In summary, matrine exerts anticancer effects by inhibiting proliferation, migration, and induces apoptosis through modulation of multiple oncogenic and tumor suppressor miRNAs across various cancer types (**Figure 3**).

### 5.6 Neferine

Neferine is a prominent bisbenzylisoquinoline alkaloid predominantly found in the seed embryo of *Nelumbo nucifera* (Gaertn.), a traditional Chinese medicine extensively utilized for the treatment of many ailments, including fever, insomnia, cardiac problems and thrombosis (136–139). Recent investigations indicated that neferine has several pharmacological actions, including anti-inflammatory, antioxidant, and antitumor properties (140–143). For example, neferine has been documented to inhibit the proliferation of human breast cancer and nasopharyngeal carcinoma cells through the modulation of miRNA levels. In MDA-MB-231 breast cancer cells, neferine demonstrated anticancer efficacy by reducing proliferation, invasion, migration, and inducing apoptosis. Neferine mediated anticancer effects were associated with downregulation of miR-374a, suggesting that neferine modulates miRNA to at least some extent. Subsequent analysis revealed that miR-374a enhances FGFR-2 expression, positioning this miRNA as an upstream regulator of a critical growth signaling network. The restoration of FGFR-2 levels mitigated neferine’s effects, indicating the reactivation of the PI3K/AKT and MEK/ERK signaling pathways (90).

**Figure 4:**
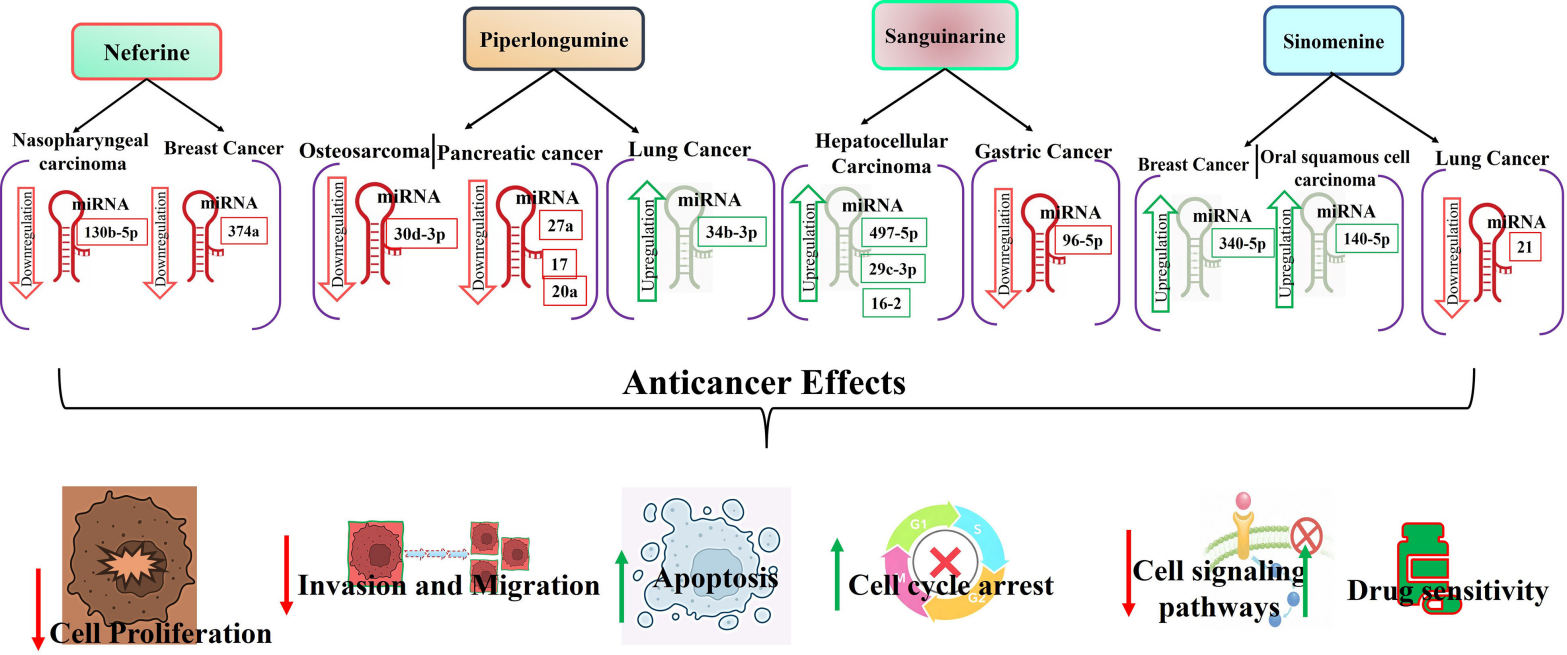
Anticancer effects of neferine, piperlongumine, sanguinarine and sinomenine by targeting miRNA across various cancer types. These alkaloids modulate the expression of oncogenic and tumor suppressive miRNAs, leading to inhibition of various oncogenic processes including cell proliferation, invasion and migration. Additionally, these alkaloids induce cell cycle arrest, apoptosis, enhance drug chemosensitivity and modulates key cell signaling pathways associated with tumor progression.

In nasopharyngeal carcinoma, neferine treatment diminished cell viability and chemoresistance and inhibited EMT characteristics in Taxol-resistant cells. Microarray analysis revealed thatmiR-130-5p is consistently downregulated after neferine treatment. Moreover, restoring miR-130b-5p levels enhanced migration and invasion while mitigating neferine’s suppressive effects on EMT driven metastasis and chemoresistance. The data suggest that neferine improves chemosensitivity and partially restricts metastatic characteristics by modulating miR-130b-5p (91). Across preclinical cancer models, neferine exerts anticancer and chemosensitizing effects by downregulating oncomiRs, thereby inhibiting EMT, metastasis and alter cell signaling pathways (**Figure 4**).

### 5.7 Piperlongumine

Piperlongumine is an alkaloid derived from the plant species *Piper longum* Linn, showing a wide array of pharmacological activities, including antitumorigenic properties in cancer cell lines and animal models (144–146). This phytochemical can specifically target and eliminate cancer cells by activating various molecular and cellular pathways associated with apoptosis. Recent studies on the anticancer properties of piperlongumine have shown that it modulates various cancer linked signaling pathways, including NF-κB, PI3K/AKT, and cyclooxygenase-2 (COX2) (147, 148). A preclinical study in lung cancer showed that piperlongumine demonstrated selective antitumor activity by inhibiting cell proliferation and inducing apoptosis. While no cytotoxicity was observed in normal epithelial cells. This effect was mediated by the upregulation of miR-34b-3p, which subsequently suppressed its target TGFBR1, thereby disrupting pro-survival signaling (92).

Piperlongumine suppressed the progression of osteosarcoma by reducing miR-30d-5p levels. The downregulation alleviates SOC3 inhibition, leading to its augmented expression and subsequent deactivation of the JAK2/STAT3 signaling pathway. This leads to increased apoptosis and decreased tumor cell proliferation, migration, and invasion. The overexpression of miR-30d-5p counteracts these effects, underscoring its critical function in facilitating the anticancer activity of piperlongumine (93).

Karki et al. demonstrated that piperlongumine suppresses proliferation and promotes apoptosis in different cancer cell lines by inducing ROS generation. It inhibits oncogenic transcription factors (Sp1, Sp3, SP4) and their target genes c-Myc via a ROS-dependent mechanism. The reduction of c-Myc resulted in the downregulation of oncomiRs (miR-27a, miR-20a, and miR-17) and the overexpression of transcriptional repressors ZBTB10 and ZBTB4 in pancreatic cancer cells following exposure to piperlongumine. This cascade ultimately diminishes Sp-driven gene expression, hence improving its anticancer efficacy (94) (**Figure 4**).

### 5.8 Sanguinarine

Sanguinarine is an alkaloid phytochemical characterized by a benzophenanthridine polycycle framework. It is primarily isolated from plant species belonging to the poppy and Fumaria (149). This compound has widespread pharmacological properties, such as antimicrobial, anti-inflammatory, antitumor, and antiangiogenesis (150–153). Sanguinarine has been reported to affect cervical cancer by influencing the ferroptosis pathway of cancer cells (154). Moreover, sanguinarine regulates the effect of tumor-associated macrophages on angiogenesis in lung cancer (155). Studies on hepatocellular carcinoma have shown that sanguinarine influences cancer cells by modulating ROS dependent mitosis and apoptosis (156). Sanguinarine exhibits strong anticancer activity in gastrointestinal malignancies by regulating key miRNAs and their downstream pathways. In gastric cancer, sanguinarine suppresses tumor growth by reducing oncogenic miR-96-5p and miR-29c-3p, leading to activation of MAPK4 and the MAPK/JNK signaling cascade (95). In hepatocellular carcinoma, it enhances tumor-suppressive miR-497-5p to inhibit CDK4-driven proliferation and also induces p53-dependent upregulation of miR-16, resulting in reduced expression of targets such as Bcl-2 and cyclin D1 (96, 97). Collectively, these findings highlight sanguinarine as a potent miRNA-modulating agent that inhibits cancer progression through coordinated control of cell cycle, apoptosis, and signaling pathways (**Figure 4**).

### 5.9 Sinomenine

Sinomenine is a benzylisoquinoline alkaloid isolated from *Sinomenium acutum* (157). Prior research has demonstrated that sinomenine possesses anti-inflammatory and anti-rheumatic properties (158, 159). Moreover, sinomenine effectively inhibits proliferation and promotes apoptosis in human cancer cell lines, including breast, lung, gastric, liver and glioblastoma (160–164). Sinomenine additionally inhibits the migration and invasion of several human cancer cells (165, 166). Recent research indicates that sinomenine possesses anticancer properties by regulating several miRNAs. In this context, sinomenine repressed the cell growth, migration, invasion and EMT of lung cancer cells by downregulating oncogenic miR-21. Treatment with sinomenine results in downregulation of MMP-2/9 and EMMPRIN, while RECK and TIMP expression is upregulated in A549 and H1299 cells. It also decreases extracellular matrix degradation and lung cancer cell invasion while enhancing epithelial traits, including E-cadherin expression (98).

Song et al. reported that sinomenine inhibits migration, vasculogenic mimicry, and EMT in hypoxic breast cancer stem-like cells through the upregulation of miR-340-5p. It further targets and suppressed SIAH2, resulting in the inhibition of the SIAH2/HIF-1α signaling pathway. This mitigates hypoxia-driven tumor advancement and diminishes aggressive cancer characteristics. Inhibition of miR-340-5p or overexpression of SIAH2 partially mitigates these effects, thereby validating the miRNA-dependent mechanism (99). In oral squamous cell carcinoma, sinomenine augments the anticancer efficacy of 5-FU through the upregulation of tumor-suppressive miR-140-5p. This miRNA directly targets PDK1, thereby suppressing anaerobic glycolysis and increasing sensitivity to 5-FU, especially in resistant cells. Sinomenine counteracts glycolytic activity and drug resistance via this miRNA-mediated pathway, whereas the inhibition of miR-140-5p attenuates theses effects in osteosarcoma both *in vitro* and *in vivo* (100) (**Figure 4**).

### 5.10 Sophocarpine

Sophocarpine, a tetracyclic quinolizidine alkaloid, is among the most prevalent bioactive constituents of *Sophora alopecuroides* L. Prior research has demonstrated that sophocarpine has several pharmacological effects, including immunoregulatory, anti-inflammatory, and anticancer properties (167–169). Recent studies indicated that sophocarpine exhibits anticancer properties against multiple types of carcinomas including, cervical, colorectal, lung, prostate, and liver cancers (170, 171). Sophocarpine exerted its anticancer effects by enhancing antitumor immunity, inducing apoptosis, and modulating various oncogenic signaling pathways (172, 173). A study by Liu et al. showed that sophocarpine induces anticancer effects in head and neck squamous cell carcinoma by modulating miR-21 activity. Sophocarpine repressed cell growth, migration, and invasion by disrupting Dicer-dependent maturation of miR-21, thereby reducing its expression. This downregulation reinstates tumor-suppressive signaling pathways which involves increased PTEN expression and p38 MAPK activation in these cancer cells. *In vivo* results also showing that sophocarpine suppressed tumor development without significant toxicity, emphasizing its promise as a miRNA-targeted therapeutic agent in head and neck carcinoma (101).

**Figure 5:**
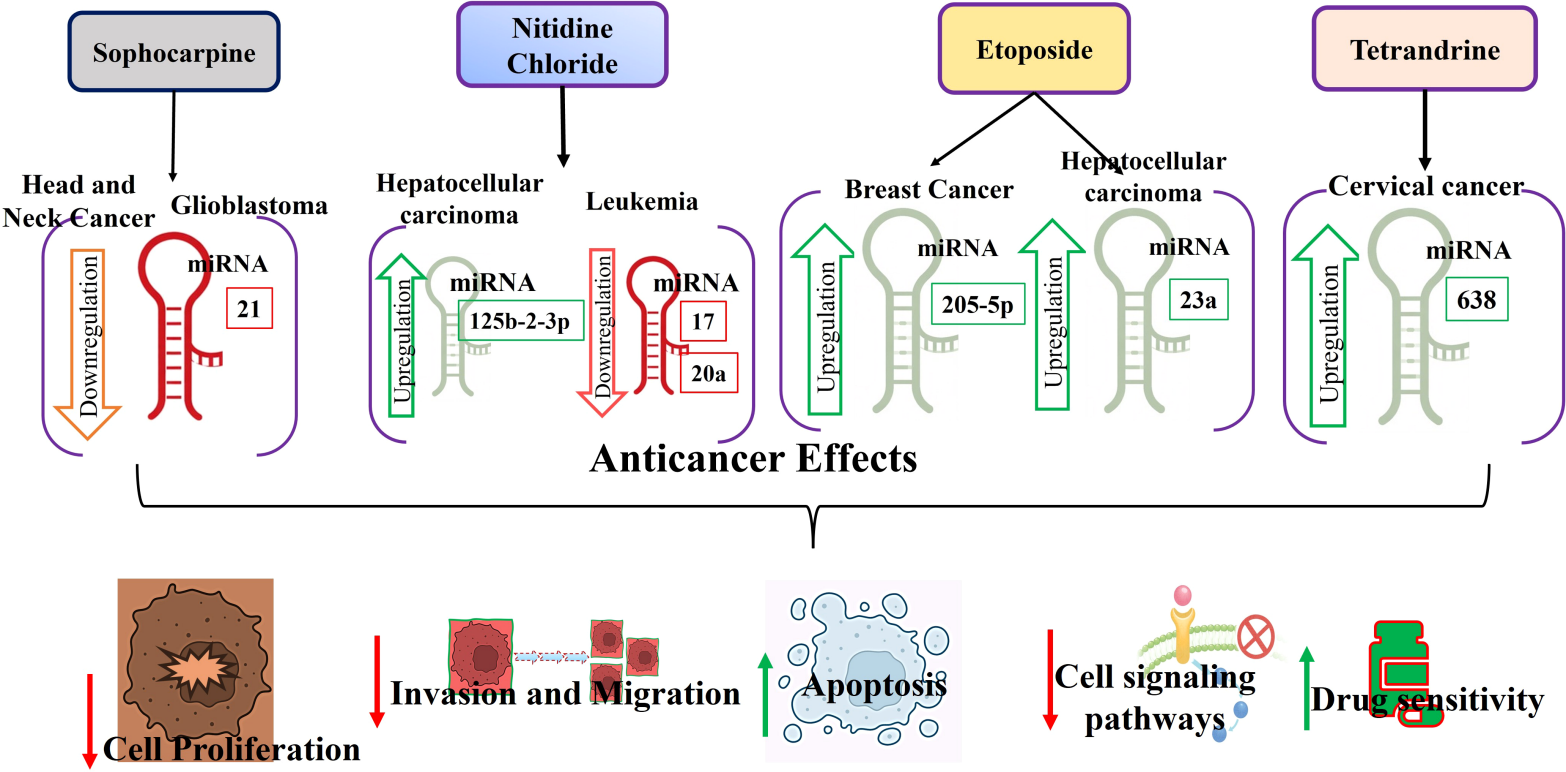
Anticancer effects of sophocarpine, nitidine chloride, etoposide and tetrandrine by targeting miRNA across various cancer types. These alkaloids modulate the expression of oncogenic and tumor suppressive miRNAs, leading to inhibition of various oncogenic processes including cell proliferation, invasion and migration. Additionally, these alkaloids induce cell cycle arrest, apoptosis, enhance drug chemosensitivity and modulates key cell signaling pathways associated with tumor progression.

Si et al., showed that sophocarpine demonstrates promising anticancer effects in glioblastoma via downregulating miR-21. Sophocarpine reduces cell growth, promoted cell cycle arrest at G0/G1 phase, and induces apoptosis. These effects are linked to the downregulation of miR-21, upregulation of PTEN, and alterations in its downstream signaling pathways. Moreover, sophocarpine inhibits the PI3K/Akt signaling pathway, hence significantly suppresses the viability of glioblastoma cells. The findings suggested that sophocarpine represses glioblastoma growth primarily by downregulating miR-21, with some involvement of PTEN signaling (102) (**Figure 5**).

### 5.11 Nitidine chloride

Nitidine chloride, extracted from *Zanthoxylum nitidum*, has been recognized as a prospective antitumor agent in many malignancies, including breast cancer, hepatocellular carcinoma, nasopharyngeal carcinoma and renal cancer (174–177). These findings indicated that nitidine chloride inhibited the proliferation of numerous cancer cells *in vitro* and *in vivo* by triggering G2/M cell cycle arrest via the suppression of the cyclin B1 and p53-mediated signaling pathways (178–180). Nitidine chloride has been documented to induce apoptosis in renal cancer cells through the ERK-associated signaling pathway, characterized by the overexpression of Bax and downregulation of Bcl-2 (181). Moreover, nitidine has been identified as a modulator of cell invasion and migration in breast and renal carcinoma cells via the AKT and c-Src-Fak signaling pathways (182, 183). Recent research indicates that nitidine chloride may modulate STAT3 and VEGF levels, which are essential components in tumor angiogenesis. Nitidine chloride has been demonstrated to be an effective chemosensitizer for various cancers (184). A study by Liu et al. demonstrates that nitidine chloride exhibits anticancer efficacy in chronic myeloid leukemia via miRNA-mediated mechanisms associated with c-Myc regulation. Nitidine chloride facilitates erythroid differentiation and triggers apoptosis, coupled with c-Myc inhibition through increased phosphorylation and degradation. Nitidine chloride reduces the expression of c-Myc-regulated oncogenic miR-17 and miR-20a, which are crucial for the survival and proliferation of leukemic cells. The restoration of c-Myc or these miRNAs mitigates the anticancer effects of nitidine chloride. Nitidine chloride also increases sensitivity to imatinib and retains efficacy in resistant cells, suggesting its potential to overcome drug resistance via miRNA-modulated pathways in leukemia (103).

A separate study shows that miR-125b-2-3p functions as a tumor suppressor in hepatocellular carcinoma and is significantly linked to clinical outcomes. Its reduced expression in hepatocellular carcinoma is associated with poor differentiation, advanced disease stage, and an unfavorable prognosis, highlighting its utility as a diagnostic and prognostic biomarker. Notably, nitidine chloride treatment enhanced the expression of miR-125b-3p which subsequently inhibited its target gene PRKCA. These data suggested that nitidine chloride facilitated restoration of tumor suppressive miRNAs enhances its therapeutic efficacy in hepatocellular carcinoma (104) (**Figure 5**).

### 5.12 Etoposide

Etoposide is a semisynthetic derivative of podophyllotoxin, a compound found naturally in mayapple tress. It possesses the chemical formula C_29_H_32_O_19_. The four-ring structure of etoposide is characterized by a lactone (a cyclic ester) and a glycosidic bond linking a glucose molecule (185). It has been a crucial component of chemotherapies for the treatment of several carcinomas, including lung cancer, lymphoma, and leukemia (186). Similar to may neoplastic agents, etoposide exhibits restricted efficacy as a monotherapy in cancer treatment (187). It is primarily linked to the combined treatment of cisplatin, carboplatin, and cyclophosphamide (188–190).

Etoposide exerts anticancer effects in breast cancer cells by modulating miR-205-5p expression. Etoposide suppresses cell proliferation, migration, and spheroid formation by upregulating miR-205-5p. Increased levels of miR-205-5p further enhance anticancer effects by directly targeting the crucial growth regulator ERBB4. These findings indicated that miR-205-5p enhances the anticancer efficacy of etoposide by inhibiting oncogenic signaling pathways (105). The study by Wang et al. reveals that etoposide treatment in hepatocellular carcinoma induces the expression of miR-23a and enhances sensitivity towards anticancer drugs. miR-23a overexpression results in diminished topoisomerase activity and disrupted S-phase progression by targeting TOP1. This effect is specific to TOP2A-targeting drugs and does not apply to chemotherapeutic drugs such as 5-FU. Conversely, upregulated levels of miR-23a diminished susceptibility to TOP1 inhibitors like irinotecan, suggesting a dual function in drug response. Moreover, miR-23a is modulated by p53 and is elevated in response to DNA damage, indicating its role in preserving topoisomerase equilibrium (106). (**Figure 5**).

### 5.13 Tetrandrine

Tetrandrine is a natural compound initially derived from Chinese herbal medicines. It is a member of the bisbenzylisoquinoline alkaloid family and is mainly derived from the root of *Stephania tetrandra* S Moore. It has been shown to possess wide range of pharmacologically significant properties (191). Tetrandrine has been utilized as a therapeutic medicine in China for decades to cure patients with hypertension, autoimmune disorders, inflammatory lung diseases, and cardiovascular diseases (192–195). Numerous preliminary studies have identified the pharmacological potential of tetrandrine in cancer therapy. Tetrandrine has been shown to inhibit cancer cell proliferation and induce apoptosis in multiple cancer cell types (196–199). It affects not only cancer cell lines but also primary tumor cells derived from ascites and pleural fluids of patients with liver, lung, gastric, and colon cancers (200). Tetrandrine exhibits several anticancer mode of action including reversal of multidrug resistance, increased tumor cell sensitivity to radiation therapy, and suppression of angiogenesis and metastasis (201–203). In this context, Wang et al. recently examined the anticancer effects of tetrandrine on cervical cancer cells via regulating miR-638 and its target gene NCAPG2. The researchers found that tetrandrine reduced the cell proliferation, invasion, migration and EMT of cervical cancer cells (HeLa and SiHa) by increasing miR-638 levels (**Figure 5**). Conversely, NCAPG2 expression decreased after tetrandrine treatment, unlike miR-638, suggesting that NCAPG2 promotes cancer growth. The findings indicate that tetrandrine prevents cervical cancer progression by augmenting miR-638 activity, which subsequently downregulates NCAPG2. This study highlighted a prospective therapeutic avenue encompassing tetrandrine and miRNA modulation in cervical cancer (107).

## 6 Limitations

Even with encouraging preclinical results of alkaloids in regulating miRNA expression, issues such as low bioavailability, rapid metabolism limited clinical trials, heterogeneity among cancer types still require attention. The diversity and structural complexity of alkaloids make their isolation and identification even more challenging, which in turn increases the time and expense needed for research and development (204). In addition to composition complexities, bioavailability and pharmacokinetic challenges are likely the most significant obstacles to clinical translation. A significant number of natural compounds exhibit inadequate water solubility, chemical instability inside the gastrointestinal tract, substantial first-pass metabolism, or fast systemic elimination (205). Furthermore, the challenge in producing successful pharmaceutical formulations is to deliver these substances safely with minimal adverse effects. Moreover, toxicity issues further complicate the process, since improper doses or delivery routes may lead to negative side effects (206). The application of nanotechnology in the formulation of natural compounds is expected to overcome a variety of difficulties in developing medicines, such as first-pass metabolism, controlled release and site-specific targeted drug delivery (207, 208). Nanocarriers, including liposomes, dendrimers, and polymer-based nanoparticles can enhance the pharmacokinetics of natural compounds and facilitate novel therapeutic strategies for chronic diseases such as cancer (209). Moreover, rationally designed clinical studies and the integration of patient specific miRNA profiling could confirm their therapeutic potential and reduce variability by enabling more customized and effective treatment options.

## 7 Conclusion and future perspectives

Recent research has shown that miRNA regulation can control tumor-suppressing genes and oncogenes. Studies conducted in preclinical cancer models has also shown its potential to impede tumor progression. miRNA profiling revealed that miRNA levels in cancer cells differ from those in healthy cells. In cancer cells, tumor-suppressing miRNAs are downregulated, whereas oncomiRs are upregulated compared with normal healthy cells. By inhibiting the activity of oncomiRs and promoting the expression of tumor-suppressing miRNAs by natural compounds, carcinogenesis and tumor growth can be partially regulated. The current review article highlighted the role of structurally diverse alkaloids to modulate miRNA expression across several cancer types by influencing cell proliferation, EMT, drug sensitivity and tumor migration. Even with these preclinical evidences, future studies are vital to assess the clinical efficacy of these anticancer effects and substantiate alkaloids as a miRNA-targeting cancer therapy. The multitargeted approach of alkaloids may include modulating the expression of cancer related miRNAs, which would enhance their complex anticancer activity and offer an additional strategy to existing cancer therapy.
